# Interactions between αCaMKII and calmodulin in living cells: conformational changes arising from CaM -dependent and -independent relationships

**DOI:** 10.1186/1756-6606-6-37

**Published:** 2013-08-19

**Authors:** Ken-ichi Kato, Taku Iwamoto, Satoshi Kida

**Affiliations:** 1 Department of Bioscience, Faculty of Applied Bioscience, Tokyo University of Agriculture, Tokyo 156-8502, Japan; 2 Core Research for Evolutional Science and Technology, Japan Science and Technology Agency, Saitama 332-0012, Japan

**Keywords:** CaMKII, Calmodulin, Fluorescence resonance energy transfer (FRET), Imaging

## Abstract

**Background:**

αCaMKII plays central and essential roles in long-term potentiation (LTP), learning and memory. αCaMKII is activated via binding with Ca^2+^/CaM in response to elevated Ca^2+^ concentration. Furthermore, prolonged increase in Ca^2+^ concentration leads to the auto-phosphorylation of αCaMKII at T286, maintaining the activation of αCaMKII even after Ca^2+^/CaM dissociation. Importantly, the active form of αCaMKII is thought to exhibit conformational change. In order to elucidate the relationships between the interaction of αCaMKII with CaM and the conformational change of αCaMKII, we generated molecular probes (YFP-αCaMKII with CFP-CaM and YFP-αCaMKII-CFP) and performed time-lapse imaging of the interaction with CaM and the conformational change, respectively, in living cells using FRET.

**Results:**

The interaction of YFP-αCaMKII with CFP-CaM and the conformational change of YFP-αCaMKII-CFP were induced simultaneously in response to increased concentrations of Ca^2+^. Consistent with previous predictions, high levels of Ca^2+^ signaling maintained the conformational change of YFP-αCaMKII-CFP at the time when CFP-CaM was released from YFP-αCaMKII. These observations indicated the transfer of αCaMKII conformational change from CaM-dependence to CaM-independence. Furthermore, analyses using αCaMKII mutants showed that phosphorylation at T286 and T305/306 played positive and negative roles, respectively, during in vivo interaction with CaM and further suggested that CaM-dependent and CaM-independent conformational changed forms displays similar but distinct structures.

**Conclusions:**

Importantly, these structual differences between CaM-dependent and -independent forms of αCaMKII may exhibit differential functions for αCaMKII, such as interactions with other molecules required for LTP and memory. Our molecular probes could thus be used to identify therapeutic targets for cognitive disorders that are associated with the misregulation of αCaMKII.

## Background

Alpha-Ca^2+^/calmodulin-dependent protein kinase II (αCaMKII), a brain-specific isoform of CaMKII, is a serine/threonine kinase [1-7].

A large number of studies, including several focused upon mouse genetics, have shown that αCaMKII plays essential and central roles in long-term potentiation (LTP), learning, memory [8-12] and emotional behavior [13,14]. αCaMKII contains an N-terminal catalytic domain that is a central regulatory domain including auto-inhibitory and calmodulin (CaM) binding regions, and a C-terminal association domain essential for the formation of a αCaMKII multi-complex. In the basal state, αCaMKII is inactive owing to intra-molecular binding of the auto-inhibitory domain to the catalytic domain [15,16]. In response to an increase in intracellular Ca^2+^ concentration, αCaMKII becomes active by interacting with Ca^2+^-bound CaM. Interaction of αCaMKII and CaM leads to the conformational change of αCaMKII (CaM-dependent active form) by dissociation of the auto-inhibitory domain from the catalytic domain. Furthermore, prolonged activation of αCaMKII by the interaction of αCaMKII with CaM results in the auto-phosphorylation of threonine-286 (T286) [17-25]. An important point to consider is that phosphorylation at T286 is thought to stabilize the CaM-bound form of αCaMKII (CaM-independent active form) and therefore, prevents the inactivation of αCaMKII kinase activity even after the dissociation of CaM [26,27]. Thus, αCaMKII functions as a “memory molecule” for Ca^2+^ signaling pathways by creating a CaM-independent (T286-phosphorylated) active form from a CaM-dependent active form in order to maintain kinase activity. In contrast, phosphorylation at T305 and T306 play an inhibitory role in the interaction with CaM [28,29]. Consequently, interactions with CaM, along with auto-phosphorylation, appear to play key regulatory roles for αCaMKII activation.

Recent studies using a fluorescence resonance energy transfer (FRET)-based technique generated a molecular probe to detect the conformational change of αCaMKII by fusing YFP and CFP to the N- or C- terminus of αCaMKII, respectively, and have shown that conformational change of αCaMKII was visualized and monitored in living neurons and single dendritic spines [30-34]. However, relationships between the interaction of αCaMKII with CaM, and the conformational change of αCaMKII, especially in regulating the switch from CaM-dependent to -independent active forms upon changes in Ca^2+^ concentration, within living cells remain unclear. In order to understand the molecular dynamics of αCaMKII activation in living cells, the present study attempted to investigate the time-dependent activation of αCaMKII following the application of drugs to HeLa cells, SH-SY5Y cells and cortical neurons. FRET was used to monitor both the interaction of αCaMKII with CaM, and also the conformational change of αCaMKII.

## Results and discussion

### Characterization of αCaMKII fusion proteins

**Figure 1:**
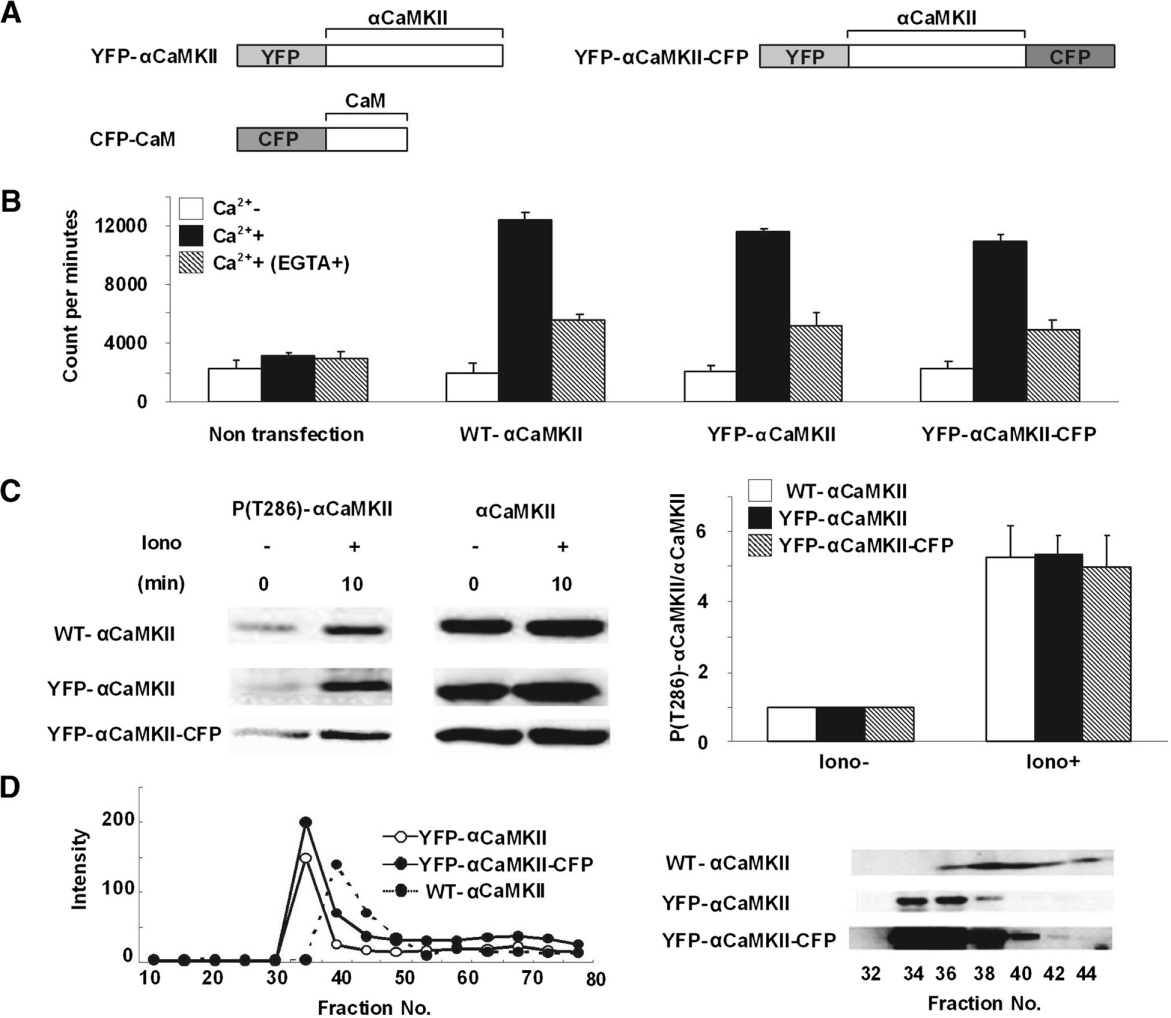
**Characterization of αCaMKII fusion proteins. (A)** Schematic representation of αCaMKII fusion proteins. **(B)** Kinase activities were measured using [γ-^32^P]-ATP incorporated into autocamtide-2 using extracts of COS-1 cells expressing WT-αCaMKII or αCaMKII fusion proteins in the absence or presence of Ca^2+^/CaM and EGTA (n = 3). **(C)** Auto-phosphorylation at T286 of αCaMKII fusion proteins. COS-1 cells expressing WT-αCaMKII or αCaMKII fusion proteins were incubated with ionomycin (0.5 μM) or DMSO and then Western blotting performed using phospho-T286 αCaMKII or αCaMKII specific antibody, respectively. The graph shows relative ratio of phospho-αCaMKII/αCaMKII level (n = 3). **(D)** Complex forming ability of αCaMKII fusion proteins. COS-1 cells expressing WT-αCaMKII or αCaMKII fusion proteins were examined by gel filtration. Left graph represents absorbance curves at 280 nm. Right panel shows Western blot analyses assaying the amount of αCaMKII fusion proteins in each fraction (fraction No. 32–44).

To monitor the interaction of αCaMKII with CaM, YFP and CFP were fused with the N-terminus of αCaMKII and CaM, respectively, resulting in the formation of YFP-αCaMKII and CFP-CaM constructs. On the other hand, to monitor the conformational changes of αCaMKII, YFP and CFP were fused with N- and C-terminus of αCaMKII, respectively, generating a YFP-αCaMKII-CFP construct (Figure 1A).

We first examined the function of our αCaMKII-fusion proteins. We showed that YFP-αCaMKII and YFP-αCaMKII-CFP exhibited comparable Ca^2+^/CaM-dependent and -independent kinase activities with WT-αCaMKII in the presence and absence of Ca^2+^/CaM with or without EGTA (Figure 1B). In addition, we showed that our αCaMKII-fusion proteins exhibited comparable levels of phosphorylation at T286 before and after the application of ionomycin (Ca^2+^ ionophore) compared to WT-αCaMKII in HeLa cells expressing WT-αCaMKII or αCaMKII-fusion proteins (Figure 1C).

The αCaMKII holoenzyme is composed of 8–12 subunits (αCaMKIIs) and we next verified the complex formation of αCaMKII fusion proteins. Gel filtration of COS-1 cell extracts expressing WT-αCaMKII or αCaMKII-fusion proteins successfully detected αCaMKII-fusion proteins (YFP-αCaMKII: 81 kDa, YFP-αCaMKII-CFP: 109 kDa) in fractions (more than 800 kDa) that exhibited larger complexes compared to WT-αCaMKII, indicating that αCaMKII-fusion proteins form a multimer (Figure 1D). Collectively, these results strongly suggested that the αCaMKII-fusion proteins used in the present study exhibited comparable function to WT-αCaMKII.

### Detecting interactions of αCaMKII with CaM and the conformational change of αCaMKII by FRET

**Figure 2:**
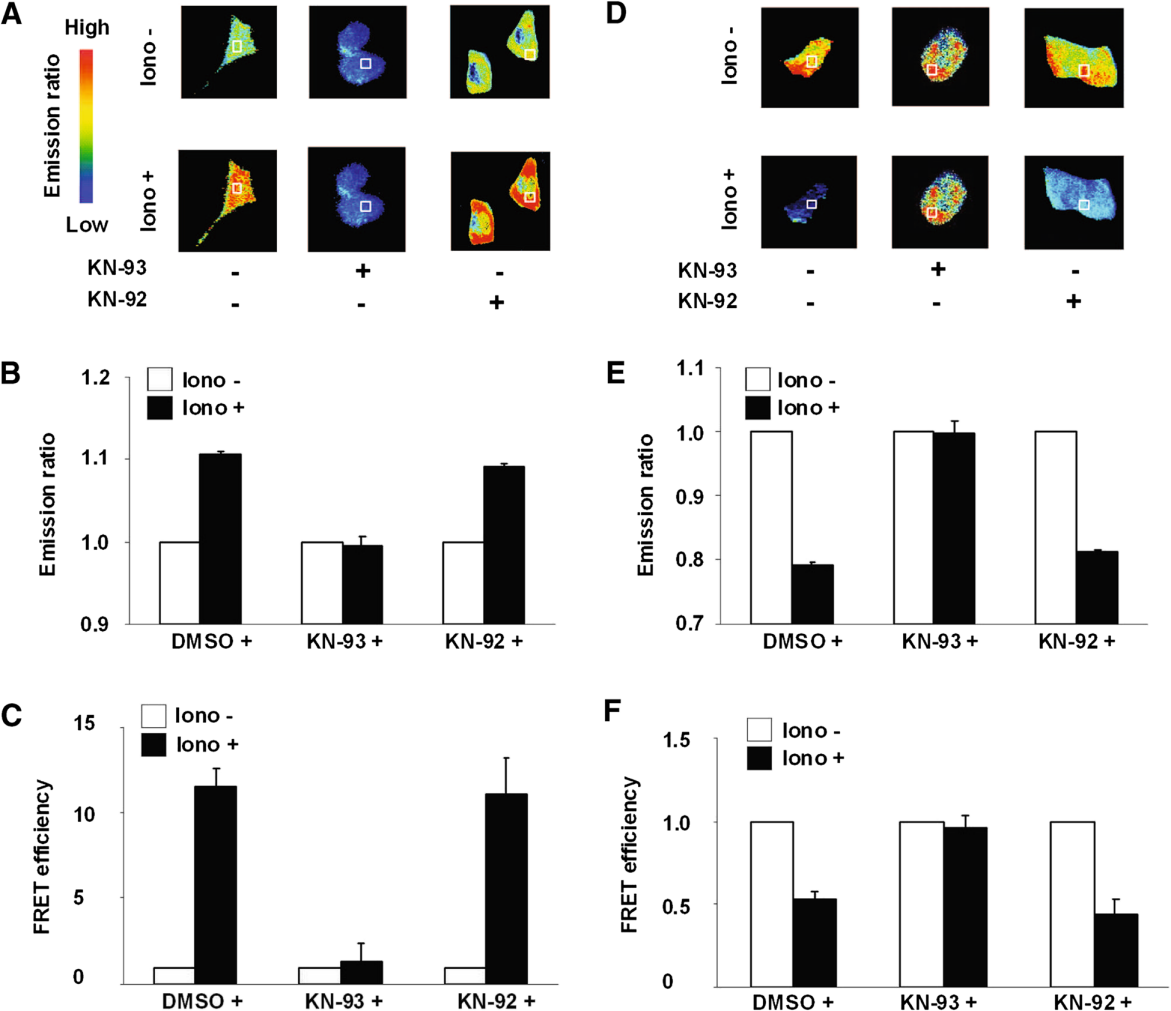
**Detection of the interaction of αCaMKII with CaM and resultant conformational changes of αCaMKII by FRET. (A, B, C)** Interaction of YFP-αCaMKII with CFP-CaM. **(D, E, F)** Conformational change of YFP-αCaMKII-CFP. **(A, D)** Images of typical YFP/CFP ratio before (−iono) and 10 min after the application of ionomycin (0.5 μM) (+iono). KN-93 (2 μM) or KN-92 (2 μM) was incubated for 30 min before the application of ionomycin. **(B, E)** Relative emission ratios before and after the application of ionomycin in the cytoplasm. 40–50 cells were analyzed in each group. **(C, F)** FRET efficiency using acceptor photo-bleaching in the cytoplasm. 20–30 cells were analyzed in each group.

A previous study examined the conformational change of αCaMKII upon changes in Ca^2+^ concentration induced by ionomycin in HeLa cells [30]. Therefore, we first tried to detect the interaction of YFP-αCaMKII with CFP-CaM induced by an increase in intracellular Ca^2+^ concentration in HeLa cells using FRET. YFP/CFP emission ratios were measured before and after the application of ionomycin in the presence or absence of KN-93, an interaction inhibitor of αCaMKII with CaM, or its inactive analog KN-92. Cells treated with ionomycin for 5 min exhibited higher emission ratios compared to non-stimulated cells (Figure 2A, B). Notably, the increase in emission ratio induced by ionomycin was blocked by KN-93, but not by KN-92 (Figure 2A, B). We next performed an acceptor photo-bleaching test [35]. The FRET efficiency of ionomycin-treated cells was higher than that of non-treated cells (Figure 2C). The observed increase in FRET efficiency was also blocked by KN-93, but not by KN-92 (Figure 2C, black bars). It is important to note that YFP-αCaMKII (1–290), that lacks the C-terminal region including CaM binding site and thereby, does not interaction with CaM [36], and CFP-CaM did not change in emission ratio and the FRET efficiency after the application of ionomycin in HeLa cells (data not shown). Taken together, these results strongly suggest that the increased emission ratio and FRET efficiency following ionomycin-application reflect the interaction of YFP-αCaMKII with CFP-CaM.

We next tried to detect the conformational change of YFP-αCaMKII-CFP in HeLa cells. Cells treated with ionomycin exhibited a lower emission ratio and FRET efficiency compared to non-treated cells. Notably, these reductions in emission ratio and FRET efficiency were blocked by KN-93, but not by KN-92 (Figure 2D–F). These results suggest that the reduced emission ratio and FRET efficiency observed following the application of ionomycin reflect the conformational change of YFP-αCaMKII-CFP arising via interaction with CaM. It is important to note that the YFP-αCaMKII-CFP used in this study exhibited similar molecular dynamics as that observed with a previous study of Camui, which also led to reductions in emission ratio and FRET efficiency in response to increased Ca^2+^ concentration [30].

Collectively, our results suggest that our molecular probes allowed us to monitor αCaMKII-CaM interaction, and αCaMKII-conformational change, following the stimulation of live cells by drugs in a real-time manner.

### Time lapse imaging of the activation of αCaMKII in HeLa cells

**Figure 3:**
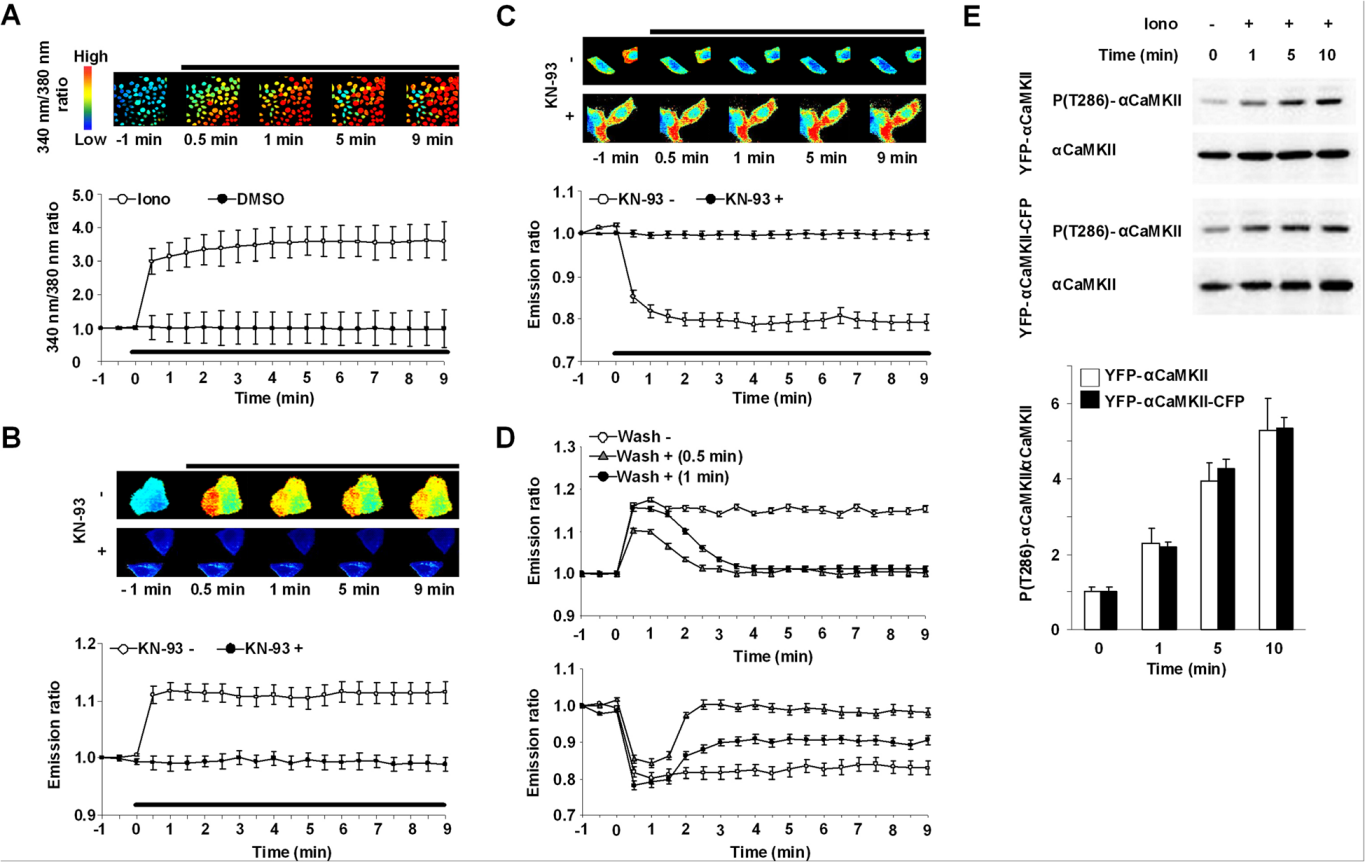
**Time lapse imaging of αCaMKII activation in HeLa cells. (A)** Changes in intracellular Ca^2+^ concentration measured using Fura2-AM. Upper panels show images of typical 340 nm/380 nm ratio at each time point before and after ionomycin application. Lower graph shows changes in relative 340 nm/380 nm ratio (open circle, ionomycin; filled circle, DMSO). Black bar shows the period of ionomycin application. 12–18 cells were analyzed in each group. **(B, C)** Changes in the interaction of YFP-αCaMKII with CFP-CaM **(B)** and the conformational change of YFP-αCaMKII-CFP **(C)**. Upper panels show images of typical YFP/CFP emission ratio before and after ionomycin application (upper images, –KN-93; lower images, +KN-93). Lower graphs show changes in relative YFP/CFP emission ratio (open circle, ionomycin; filled circle, ionomycin with KN-93). Black bar shows the period of ionomycin application. 9–13 cells were analyzed in each group. **(D)** Effect of removal of ionomycin by wash out at 0.5 or 1 min following ionomycin application. Upper graph shows the interaction of YFP-αCaMKII with CFP-CaM (open circle, –wash; triangle, 0.5 min; filled circle, 1 min). Lower graph shows the conformational change of YFP-αCaMKII-CFP (open circle, -wash; triangle, 0.5 min; filled circle, 1 min). 10–12 cells were analyzed in each group. **(E)** Auto-phosphorylation at T286 of αCaMKII fusion proteins after ionomycin application at each time point (0, 1, 5 and 10 min). Upper panel shows Western blot analyses analyzing the expression level of phospho-αCaMKII fusion proteins and total-αCaMKII fusion proteins. Lower graph shows relative ratio of phospho-αCaMKII/αCaMKII levels (n = 3).

We attempted to monitor the activation of αCaMKII in HeLa cells by time lapse imaging. Figure 3A shows changes in intracellular Ca^2+^ concentration following the application of ionomycin. The increase in emission ratio was observed within 30 sec of ionomycin application and maintained throughout the period of monitoring, indicating that ionomycin induced a long-term increase in intracellular Ca^2+^ concentration. Figure 3B, 3C show time lapse imaging (30 sec interval) of the interaction of YFP-αCaMKII with CFP-CaM and the conformational changes of YFP-αCaMKII-CFP, respectively, before and after the application of ionomycin. Similarly, emission ratios from YFP-αCaMKII with CFP-CaM and YFP-αCaMKII-CFP was maximally increased or decreased 30 sec after ionomycin application and was maintained at maximal or minimal levels thereafter, respectively (Figure 3B, C). These increased and decreased emission ratios were maintained for at least 24 min (Additional file 1: Figure S1A). Furthermore, time lapse imaging every 1 sec revealed that the emission ratio was increased or decreased within 5 sec and reached maximum or minimal levels, respectively, within just 30 sec of ionomycin application (Additional file 1: Figure S1B). Notably, pre-treatment with KN-93 abolished the changes in the emission ratio induced by the application of ionomycin (Figure 3B, C). These results indicated that the interaction of YFP-αCaMKII with CFP-CaM, and the conformational change of YFP-αCaMKII-CFP, were induced immediately after ionomycin application and continued during the period in which Ca^2+^ concentration increased. It is important to note that YFP-αCaMKII-CFP exhibited similar long-term conformational changes as observed in a previous study [30].

To further clarify the dynamic regulation of αCaMKII-activation in response to changes in Ca^2+^ concentration, we examined the effects of washing out the ionomycin. Consistent with previous observations (Figure 3B, C), the relative emission ratio from cells expressing YFP-αCaMKII and CFP-CaM began to increase immediately following the application of ionomycin (Figure 3D, upper panel). However, this increased emission ratio gradually returned to baseline levels following the removal of inonomycin. Importantly, this decreasing emission ratio curve from YFP-αCaMKII with CFP-CaM correlated with that of Ca^2+^ concentration following the removal of ionomycin (Additional file 1: Figure S1C), indicating that the interaction of YFP-αCaMKII with CFP-CaM reflects changes in intracellular Ca^2+^ concentration. Similarly, cells expressing YFP-αCaMKII-CFP displayed a transient reduction in emission ratio when ionomycin was applied for 0.5 min (Figure 3D, lower panel). However, in the case of ionomycin application for 1 min, the reduced emission ratio did not return to baseline levels and was maintained at a point that was 70% of the minimum level (Figure 3D, lower panel), even when the increased emission ratio from YFP-αCaMKII with CFP-CaM returned to the baseline. This was the case even after dissociation of CFP-CaM from YFP-αCaMKII, or after the release of CFP-CaM from YFP-αCaMKII (Figure 3D, upper panel), suggesting that YFP-αCaMKII-CFP exhibited CaM-independent conformational change during this stage. This observation supported previous predictions stating that αCaMKII exhibits CaM-independent conformational change even after the dissociation of CaM from αCaMKII when prolonged increases in Ca^2+^ concentration leads to the auto-phosphorylation of αCaMKII at T286. In contrast, a comparison of the results in Figure 3 (3B VS 3C, D upper panel VS lower panel) indicated that changes in emission ratios from YFP-αCaMKII with CFP-CaM and YFP-αCaMKII-CFP were inversely correlated especially for the first 0–5 min after ionomycin application, suggesting that YFP-αCaMKII-CFP displayed CaM-dependent conformational change during this stage.

In a manner similar to endogenous αCaMKII, auto-phosphorylation levels of YFP-αCaMKII and YFP-αCaMKII-CFP at T286 increased with time following the application of ionomycin (Figure 3E, data not shown). More importantly, when ionomycin was applied for 1 min, but not 30 sec, increased levels of T286-phosphorylation were sustained even 10 min after the removal of ionomycin (Figure 3E, data not shown), supporting our conclusion that auto-phosphorylation of YFP-αCaMKII-CFP at T286 contributed to CaM-independent conformational change after the dissociation of CaM.

### Time lapse imaging of the activation of αCaMKII in SH-SY5Y cells

**Figure 4:**
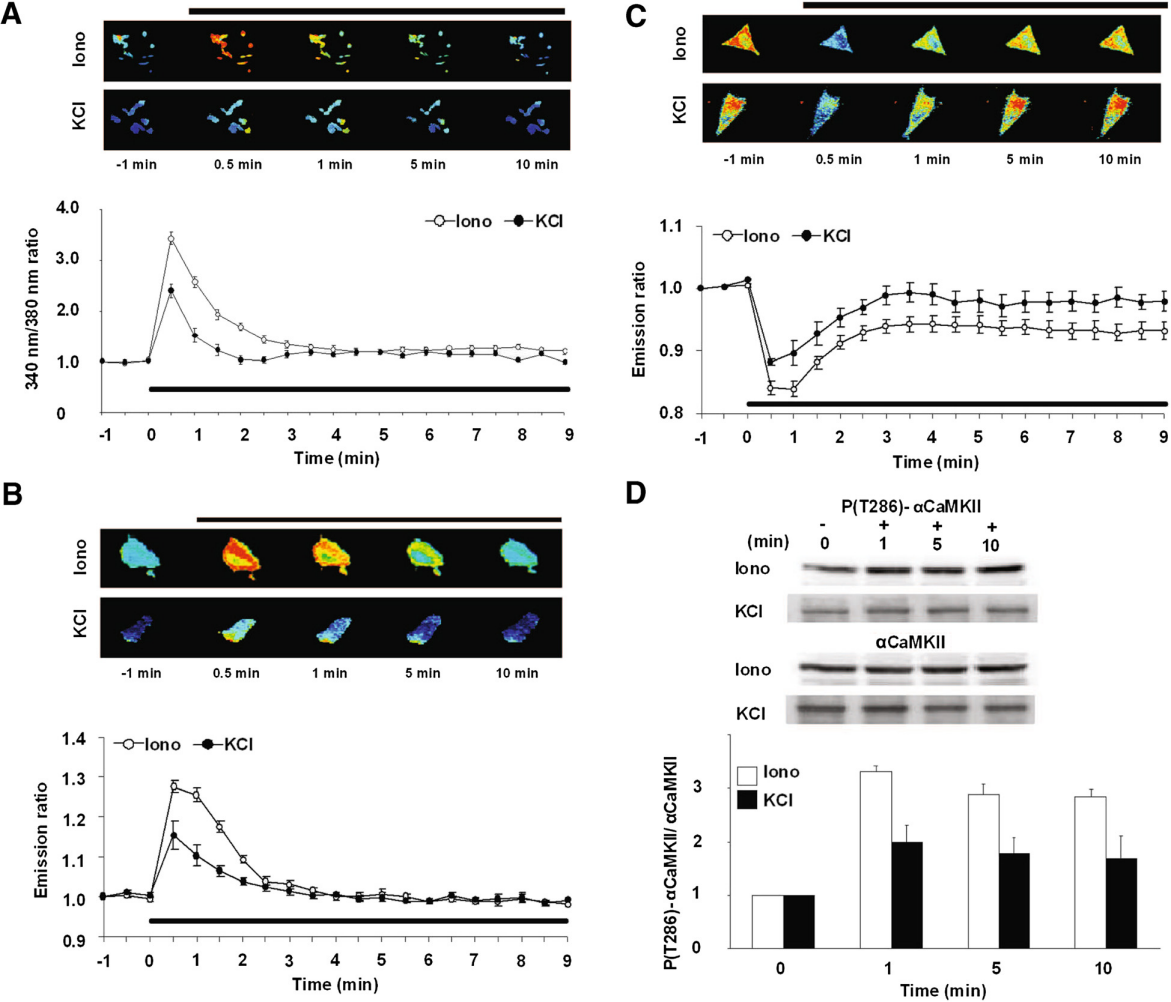
**Time lapse imaging of αCaMKII activation in SH-SY5Y cells. (A)** Changes in intracellular Ca^2+^ concentration measured using Fura2-AM. Upper panels show images of typical 340 nm/380 nm ratio at each time point before and after the application of ionomycin or KCl (50 mM)-application. Lower graph shows changes in relative 340 nm/380 nm ratio (open circle, ionomycin; filled circle, KCl). Black bar shows the period of ionomycin or KCl application. 30–40 cells were analyzed in each group. **(B, C)** Changes in the interaction of YFP-αCaMKII with CFP-CaM **(B)** and the conformational change of YFP-αCaMKII-CFP **(C)**. Upper panels show images of typical YFP/CFP emission ratio before and after ionomycin or KCl application. Lower graphs show changes in relative YFP/CFP emission ratio (open circle, ionomycin; filled circle, KCl). Black bar shows the period of ionomycin or KCl application. 11–25 cells were analyzed in each group. **(D)** Auto-phosphorylation at T286 of αCaMKII fusion proteins after ionomycin or KCI application at each time point (0, 1, 5 and 10 min). Upper panel shows Western blot analyses analyzing the expression level of phospho-αCaMKII fusion proteins and total-αCaMKII fusion proteins. Lower graph shows relative ratio of phospho-αCaMKII/αCaMKII levels (n = 3).

We next attempted to examine the dynamics of αCaMKII activation in neurons. To do this, we first imaged αCaMKII activation in SH-SY5Y cells, human neuroblastoma cells, which are referred to as having neuron-like physiology. We observed an increase in Ca^2+^ concentration within 30 sec of ionomycin application (Figure 4A). However, in contrast to the results obtained using HeLa cells, this increased Ca^2+^ concentration returned to baseline levels within 10 min. We further characterized Ca^2+^ dynamics in SH-SY5Y cells using KCl which induces an increase in Ca^2+^ concentration via depolarization in a similar manner to physiological conditions. Similarly, transient increases in Ca^2+^ concentration were observed following the application of KCl, although these levels of Ca^2+^ concentration were lower than that induced by ionomycin and returned to baseline within 3 min (Figure 4A). These results indicated that Ca^2+^ dynamics following drug application exhibited cell-type specificity.

In Figure 4B and C, we observed similar time-course changes in emission ratios from YFP-αCaMKII with CFP-CaM and YFP-αCaMKII-CFP in the presence of ionomycin to those obtained from HeLa cells treated with ionomycin for 1 min (Figure 3D); emission ratios from YFP-αCaMKII-CFP was maintained at a reduced level even after the increased emission ratios from YFP-αCaMKII with CFP-CaM returned to the baseline. These results suggest that YFP-αCaMKII-CFP exhibits CaM-dependent conformational change and then CaM-independent conformational change (Figure 4C). In contrast, when SH-SY5Y cells were treated with KCl, emission ratios from YFP-αCaMKII with CFP-CaM, and YFP-αCaMKII-CFP were either increased or decreased, respectively, but both returned to their respective baseline (Figure 4B VS 4C), suggesting that YFP-αCaMKII-CFP exhibited CaM-dependent conformational change but failed to display a CaM-independent form. Importantly, changes in emission ratios from YFP-αCaMKII with CFP-CaM, and YFP-αCaMKII-CFP following KCl application were smaller than those induced by ionomycin application (Figure 4B, C). This observation appears to reflect a lower increase of intracellular Ca^2+^ concentration by KCl compared to ionomycin (Figure 4A).

Phosphorylation of αCaMKII-fusion proteins at T286 was increased following the application of ionomycin or KCl (Figure 4D). However, the level of T286-phosphorylation in response to ionomycin was higher than that by KCl. Furthermore, the increased level of T286-phosphorylation by ionomycin was maintained, while this phosphorylation induced by KCl was kept at the lower level (Figure 4D). These observations suggest that CaM-independent conformational changes of YFP-αCaMKII-CFP led by the application of ionomycin reflect prolonged phosphorylation at T286. In contrast, the observation that the application of KCl failed to lead to CaM-independent conformational change in YFP-αCaMKII-CFP is thought to reflect the lower level and transient increase of T286-phosphorylation.

### Monitoring of the molecular dynamics of αCaMKII in living cortical neurons

**Figure 5:**
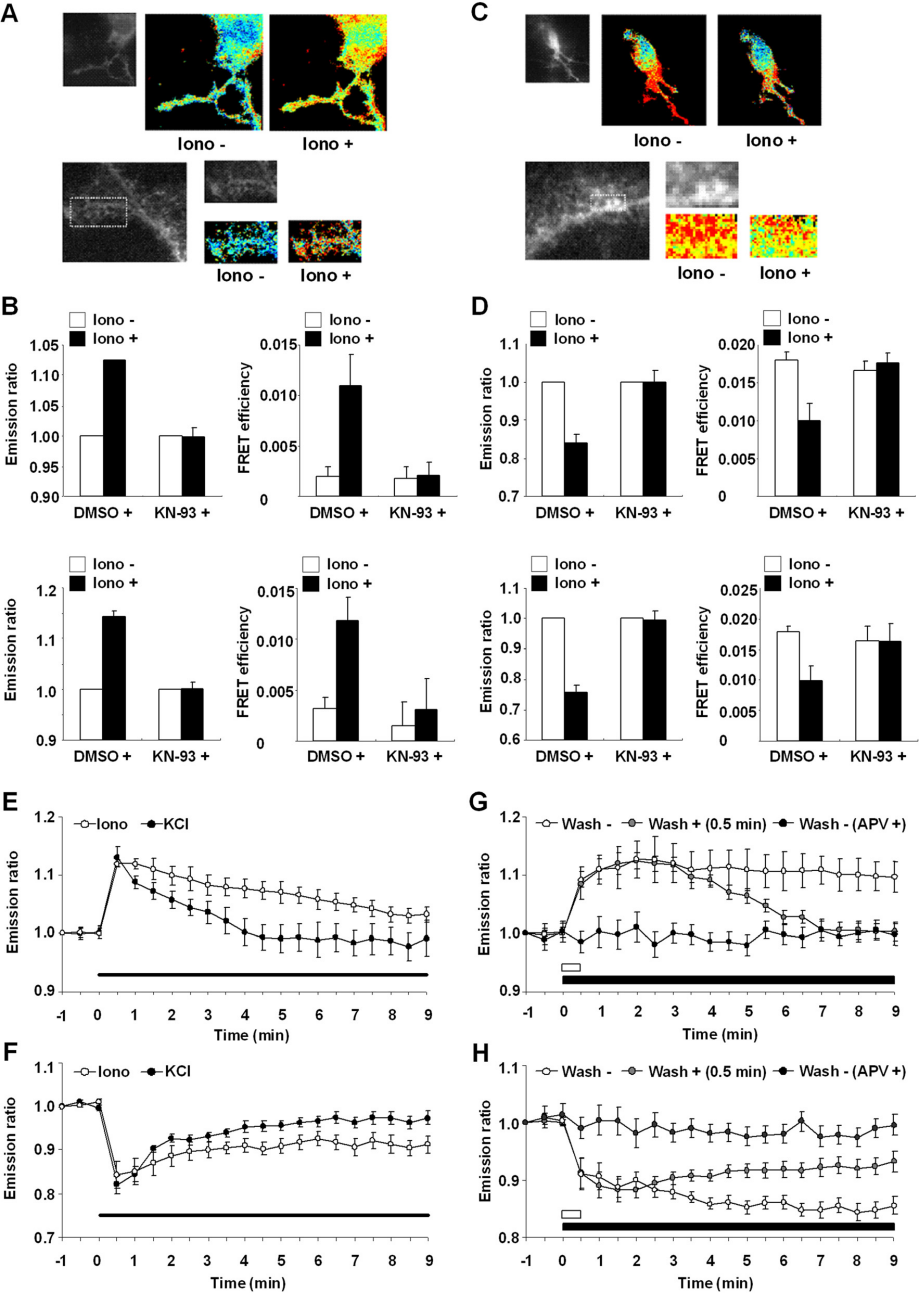
**Monitoring the molecular dynamics of αCaMKII in living cortical neurons. (A, C)** Typical images of the interaction of YFP-αCaMKII with CFP-CaM **(A)** and the conformational change of YFP-αCaMKII-CFP **(C)** in the cytoplasm (upper panels) and dendrite (lower panels) before and after ionomycin application (0.5 μM) for 10 min. Monochrome images show YFP fluorescence. **(B, D)** Left graphs show relative emission ratio and right graphs show FRET efficiencies before (white bars) and after (black bars) ionomycin application. Upper graphs show the results of cytoplasm. Lower graphs show the results of dendrites. **(E, F)** Changes in relative emission ratio following the application of ionomycin or KCl (open circle, ionomycin; filled circle, KCl). Black bar shows the period of ionomycin or KCl application. 9–10 cells were analyzed in each group. **(G, H)** Effect of short or long-term activation of NMDA receptor by the application of NMDA (25 μM) with or without pre-treatment of AP5 (25 μM) (open circle, long-term; gray circle, short-term; filled circles, long-term with pre-treatment of AP5). Black bar and white bar show the period of NMDA application. 7–9 cells were analyzed in each group.

We finally examined the dynamics of αCaMKII in living neurons. We observed similar changes in emission ratio and FRET efficiency in the soma and dendrites of cortical neurons expressing YFP-αCaMKII with CFP-CaM, or YFP-αCaMKII-CFP to those of HeLa cells (Figures 2 and 5A–D). Consequently, we performed time-lapse imaging of αCaMKII-activation in cortical neurons (Figure 5E, F). Changes in emission ratios from YFP-αCaMKII with CFP-CaM and YFP-αCaMKII-CFP were inversely correlated in the presence of ionomycin; both emission ratios changed but did not returned to baseline levels (Figure 5E, F). Thus, cortical neurons exhibited interaction of YFP-αCaMKII with CFP-CaM and conformational change of YFP-αCaMKII-CFP, following the application of ionomycin.

We next examined the molecular dynamics of αCaMKII activation upon an increase in Ca^2+^ concentration mediated by the NMDA receptor which plays essential roles in synaptic plasticity in neurons. To do this, we examined the effects of applying NMDA. As with HeLa cells (Figure 3), the long-term application of NMDA increased or decreased emission ratios from YFP-αCaMKII with CFP-CaM or YFP-αCaMKII-CFP, respectively (Figure 5G, H). These changes in emission ratios were maintained throughout imaging, suggesting that long-term NMDA-application prolonged the interaction of YFP-αCaMKII with CFP-CaM and the conformational change of YFP-αCaMKII-CFP. In contrast, when NMDA was washed out at 30 sec after application, the increased emission ratios from YFP-αCaMKII with CFP-CaM retuned to baseline. Furthermore, while the reduced emission ratio from YFP-αCaMKII-CFP was increased, it did not return to baseline levels. As with our previous observations, these results suggest that YFP-αCaMKII-CFP exhibits CaM-dependent conformational change and then CaM-independent conformational change following the removal of NMDA. Importantly, the co-application of AP5, a known blocker of the NDMA receptor, abolished the changes in emission ratios following NMDA application, indicating that changes in emission ratio observed after NMDA application were mediated by the NMDA receptor.

### Effects of mutations at T286/305/306 upon the molecular dynamics of CaMKII

**Figure 6:**
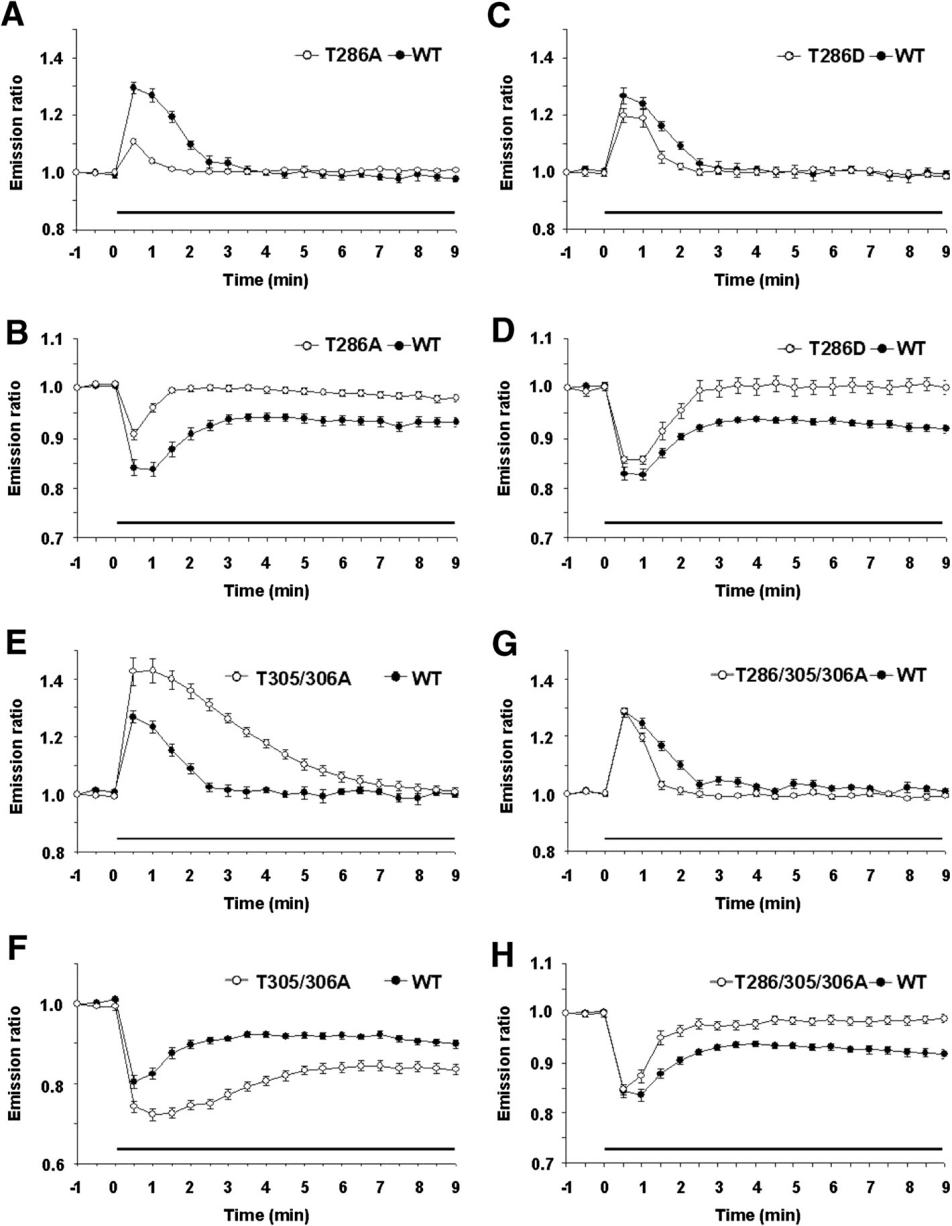
**Effects of mutations at T286/305/306 upon the molecular dynamics of αCaMKII in SH-SY5Y cells.** Time lapse imaging of αCaMKII mutants. Graphs show relative YFP/CFP emission ratio (open circle, αCaMKII mutant fusion proteins; filled circle, WT αCaMKII fusion proteins). Black bar shows the period of ionomycin application. **(A, B)** T286A. **(C, D)** T286D. **(E, F)** T305/306A. **(G, H)** T286/305/306A. **(A, C, E, G)** Interaction of αCaMKII with CaM. **(B, D, F, H)** Conformational change of αCaMKII. 20–30 cells were analyzed in each group.

We finally examined the effects of mutating αCaMKII at T286 to alanine (A) or aspartate (D) in SH-SY5Y cells. The T286A mutant can bind with CaM but is not phosphorylated at T286, whereas the T286D mutant mimics the T286-phosphorylated form and functions as a constitutive active form of αCaMKII. Emission ratios from YFP-T268A with CFP-CaM and YFP-T268A-CFP showed much smaller increases or decreases, respectively, following the application of ionomycin compared to control fusion proteins (Figure 6A, B). That emission ratios from YFP-T268A-CFP returned to the baseline was not surprising because this mutant is not phosphorylated at T286, and thus lacks the ability to undergo CaM-independent conformational change. More importantly, the observation that emission ratios from YFP-T286A with CFP-CaM exhibited much smaller reductions compared to control fusion proteins indicates that T286-phosphorylation is not only required for CaM-independent conformational change but also plays regulatory roles in the interaction with CaM and supports the hypothesis that phosphorylation at T286 stabilizes the binding of CaM with αCaMKII [26,27]. On the other hand, YFP-T286D with CFP-CaM exhibited increased emission ratios immediately following the application of ionomycin but more importantly, faster reductions in emission ratios compared to control fusion proteins, suggesting that the T286D mutation enhanced the dissociation of CaM (Figure 6C, D). It is possible that the constitutive kinase activation of the T286D mutant enhances phosphorylation at T305/306, thereby facilitating dissociation of CaM. In addition, emission ratios from YFP-T286D with CFP-CaM and YFP-T286D-CFP was simultaneously increased or decreased, respectively, following ionomycin application and then returned to baseline, indicating that the T286D mutant exhibited only CaM-dependent conformational change (Figure 6C, D). However, these observations do not mean that YFP-T286D-CFP fails to form CaM-independent (T286-phosphorylation-dependent) conformational change because it is thought that the T286D mutant had already formed similar conformation with the T286-phosphorylated form of αCaMKII at baseline levels. More importantly, the observed reduction in emission ratio from YFP-T286D-CFP following ionomycin application suggests that the interaction with CaM leads to the further conformational change of this mutant. Therefore, this observation also suggests that T286-phosphorylated αCaMKII displays similar but distinct conformation with CaM-bound αCaMKII.

We finally examined the effects of mutations at T305/306 to A. In contrast to the results obtained with T286 mutants, we observed a higher emission ratio from YFP-T305/306A with CFP-CaM compared to control fusion protein (Figure 6E, F). Since phosphorylation at T305/306 enhances the dissociation of CaM, this increased emission ratio may reflect an increased number of complexes formed by YFP-T305/306A with CFP-CaM. Consistently, lower emission ratios from YFP-T305/306A-CFP were observed compared to control fusion protein during which emission ratios from YFP-T305/306A with CFP-CaM was increased (Figure 6E, F). Interestingly, the addition of T305/306A mutations into the T286A mutant (T286/305/306A) rescued impairments in the interaction with CaM observed with the T286A mutant (Figure 6G, H), although this T286/305/306A mutant failed to exhibit CaM-independent conformational change. Therefore, this mutant is thought to exhibit normal ability to interact with CaM and to adopt CaM-dependent conformational change, but lacks CaM-independent conformational change (Figure 6G, H). It is important to note that mutant proteins exhibited comparable expression levels with YFP-αCaMKII-CFP in the presence and absence of drugs (Additional file 2: Figure S2).

αCaMKII is known to play essential roles in LTP and memory, and functions as a memory molecule enabling the maintenance of kinase activity by changing from a CaM-dependent active form to a CaM-independent (T286-phosphorylated) active form, even after Ca^2+^/CaM dissociation. In the present study, to understand the molecular dynamics of αCaMKII activation via interaction with CaM and the phosphorylation of αCaMKII, we generated YFP-αCaMKII, CFP-CaM and YFP-αCaMKII-CFP and using these probes, performed real-time imaging of both αCaMKII-CaM interaction and the conformational change of αCaMKII in living cells in response to increases in intracellular Ca^2+^ concentration. Our results indicated that changes in intracellular Ca^2+^ concentration tightly correlate with that of emission ratio from YFP-αCaMKII with CFP-CaM (Figures 3, 4 and 5); intracellular Ca^2+^ concentration and the observed emission ratios increased simultaneously following the application of drugs, indicating that the complex formation of αCaMKII and CaM depended upon the intracellular concentration of Ca^2+^. Furthermore, changes in emission ratios from YFP-αCaMKII with CFP-CaM and YFP-αCaMKII-CFP were inversely correlated with increasing intracellular Ca^2+^ levels and during periods when high intracellular Ca^2+^ concentration was maintained (Figures 3, 4 and 5), indicating that YFP-αCaMKII-CFP exhibited CaM-dependent conformational change at this stage. In contrast, decreased emission ratio from YFP-αCaMKII-CFP did not return to the baseline and was maintained at constant levels even when the emission ratios from YFP-αCaMKII with CFP-CaM returned to baseline when Ca^2+^ signaling was highly active. These observations indicate that YFP-αCaMKII-CFP exhibits CaM-independent (perhaps, T286-phosphorylation dependent) conformational change at this stage. Thus, we observed the transfer of conformational change of YFP-αCaMKII-CFP from a CaM-dependent form into a CaM-independent form when intracellular Ca^2+^ concentration was first increased to high levels and then subsequently decreased. Consequently, our current observations strongly support previous predictions that αCaMKII exhibits CaM-independent conformational change, even after the dissociation of CaM [6,17].

As described above, our findings that decreased emission ratios from YFP-αCaMKII-CFP were increased following a reduction in Ca^2+^ concentration in SH-SY5Y cells, or after drug wash out in HeLa cells and cortical neurons, but did not return to baseline suggests that the CaM-dependent conformation of YFP-αCaMKII-CFP exhibits less FRET compared to CaM-independent conformation. This observation suggests that CaM-bound and CaM-unbound (but T286-phosphorylated) active forms of αCaMKII exhibit distinct conformations, or differences in their conformation such as the distance or angle between CFP and YFP. Interestingly, YFP-T286D-CFP displayed reductions in emission ratio only during when YFP-T286D interacted with CFP-CaM. These observations strongly support our conclusion that differences in the observed emission ratios between CaM-dependent and -independent active forms of YFP-αCaMKII-CFP reflect differences in their conformation. Therefore, our observations raise the possibility that CaM-dependent and -independent active forms also exhibit differences not only in their conformation but also in their function, such as the interaction of αCaMKII with other proteins including GluN2B, which play critical roles in LTP and memory. Further studies are required to investigate functional differences between CaM-dependent and independent active forms of αCaMKII.

Mutation of T286 to an A residue resulted in reduced interaction with CaM. This observation strongly suggests that phosphorylation at T286 plays regulatory roles in the interaction with CaM, as well as CaM-independent conformational change, and supports previous predictions that phosphorylation at T286 stabilizes interactions with CaM. On the other hand, T305/306A mutations resulted in increased interaction with CaM. This observation is also consistent with previous predictions and indicates that in contrast to T286, T305/306 plays inhibitory roles in terms of CaM interactions. Notably, our observations that triple mutations at T286/305/306 counter-balanced the effects of T286A and T305/306A mutations provided significant support for our conclusions that T286 and T305/306 mutations play positive and negative roles, respectively, with respect to CaM interaction. Thus phosphorylation at T286 and T305/306 mediate the strength of Ca^2+^ signaling activation by regulating interactions with CaM and conformational change, and by determining the duration of αCaMKII-activation in vivo.

## Conclusions

In summary, we demonstrated the molecular dynamics of αCaMKII activation via the transition from CaM-dependent to -independent active forms. Future studies now required to investigate the molecular mechanisms underlying the formation of LTP and memory by monitoring αCaMKII activation in a real-time manner using the molecular probes developed in this study. Furthermore, our molecular probes could be applied to identify therapeutic targets for cognitive disorders such as Angelman syndrome which is associated with the misregulation of αCaMKII [37].

## Methods

### Plasmid constructions

The full-length cDNA encoding mouse αCaMKII was amplified by PCR using cDNA from the whole brain of C57B/6 mouse as a template with the following primers: forward primer, ggg tct aga tgt aca aga tgg cta cca tca cct gc, and reverse primer, ggg ggt acc ggg ccc atg cgg cag gac gga ggg. The resulting PCR fragment was sub-cloned into the *Xba*I-*Kpn*I sites of pBluescriptII (SK–) (Stratagene), generating pBS-αCaMKII. To generate full-length cDNA encoding T286A and D, and T305/306A, two separate fragments (nucleotides 1 to 858 and 859 to 1437, and 1 to 945 and 859 to 1437, respectively, in αCaMKII) were amplified by PCR using pBS-αCaMKII as a template with the following primers: 1/858 forward primer, ggg tct aga tgt aca aga tgg cta cca tca cct gc, and 1/858 T286A reverse primer, ggg gga tcc **gtc gac** ggc ctc ctg tct gtg cat gca, or 1/858 T286D reverse primer, ggg gga tcc **gtc gac** g**tc** ctc ctg tct gtg cat gca (mutated sequences are boxed); 859/1437 forward primer, ggg gga tcc **gtc gac** tgc ctg aag aag ttc, and 859/1437 reverse primer, ggg ggt acc ggg ccc atg cgg cag gac gga ggg; 1/945 forward primer, ggg tct aga tgt aca aga tgg cta cca tca cct, and 1/945 reverse primer, ggg gaa ttc **tcc gga** gaa gtt cct ggt ggc cag cat cag cag gag gat ggc tcc ctt cag (mutated sequences are boxed); 945/1437 forward primer, ggg gga tcc **tcc gga** ggg aag agc gga, and 945/1437 reverse primer, ggg ggt acc ggg ccc atg cgg cag gac gga ggg. To ligate the two fragments, we made use of *Sal*I or *Bsp*EI restriction sites that we had introduced at the 3′ and 5′ ends of the coding region by amplification with the above primers (see sequences in bold). The full length T286A, T286D or T305/306A mutant sequences comprising the two ligated PCR fragments were inserted into the *Xba*I-*Kpn*I sites of pBluescriptII (SK–), generating pBS-T286A, pBS-T286D and pBS-T305/306A, respectively. The *Xba*I-*Apa*I fragment encoding WT-αCaMKII from pBS-αCaMKII was subsequently sub-cloned into pcDNA3 (Invitrogen), generating a plasmid expressing WT-αCaMKII (pWT-αCaMKII). The *Bsr*GI-*Apa*I fragment encoding αCaMKII from pBS-αCaMKII was sub-cloned into pEYFP-C1 (CLONTECH), generating a plasmid expressing YFP-αCaMKII (pYFP-αCaMKII). The full-length cDNA encoding CFP was amplified by PCR using pECFP-C1 (CLONTECH) as a template with the following primers: forward primer, ggg ggt acc ggg ccc atg gtg agc aag ggc, and reverse primer, ggg gga tcc tca ctt gta cag ctc gtc cat. The resulting PCR fragment was then sub-cloned into the *Apa*I-*Bam*HI sites of pYFP-αCaMKII, generating a plasmid expressing YFP-αCaMKII-CFP (pYFP-αCaMKII-CFP). Using similar procedures, pYFP-T286A, pYFP-T286D, pYFP-T305/306A, pYFP-T286A-CFP, pYFP-T286D-CFP and pYFP-T305/306A-CFP were generated, respectively. Using similar procedure with that of mouse αCaMKII, the full-length cDNA encoding mouse CaM was amplified by PCR as a template with the following primers: forward primer, ggg tcc gga atg gct gat cag ctg act, and reverse primer, ggg gaa ttc ttt tgc agt cat cat ctg. The resulting PCR fragment was subcloned into the *Bsp*EI-*Eco*RI sites of pECFP-C1, generating a plasmid expressing CFP-CaM (pCFP-CaM). To generate the plasmids expressing YFP-T286/305/306A or YFP-T286/305/306A-CFP (pYFP-T286/305/306A and pYFP-T286/305/306A-CFP), the *Bsr*GI-*Sal*I fragment encoding T286A from pYFP-T286A was sub-cloned into pYFP-T305/306 or pYFP-T305/306A-CFP, respectively.

### Cell culture and transient transfection

As described previously [38,39], cultured cells were maintained at 37°C in 95% O_2_ 5% CO_2_ in Dulbecco’s modified Eagle’s medium (DMEM, NISSUI) supplemented with penicillin (100 U/ml) streptomycin (100 mg/ml) and 5% (HeLa cells and COS-1 cells) or 10 % (SH-SY5Y cells) fetal bovine serum (FBS, JRH BIOSCIENCE), respectively, and transiently transfected. Similarly, rat brain cortical neurons (CAMBREX) were maintained in Neuro basal medium (Invitrogen) supplemented with Penicillin-Streptomycin Mixture (TaKaRa), 200 mM L-Glutamine and B27 Supplement (Invitrogen). Rat cortical neurons or, HeLa cells, COS-1 cells or SH-SY5Y cells grown in a 35 mm glass-bottom culture dish with or without coating by poly-D-lysine (Mat Tek) were transiently transfected with pYFP-αCaMKII (3 or 1 μg) and pCFP-CaM (3 or 1 μg), or pYFP-αCaMKII-CFP (6 or 2 μg) using Lipofectamine™ and Nupherin™ (BIOMOL international) or PLUS™ Reagent and Lipofectamine™ (Invitrogen), respectively. Solutions of drugs were dissolved in DMSO at 1 mM [ionomycin (SIGMA), KN-93 (CALBIOCHEM), KN-92 (CALBIOCHEM) and AP5 (SIGMA)], or water at 1 M (KCl) or 100 mM [NMDA (SIGMA)]. These stock solutions were diluted into Hanks Balanced Salt Solution (HBSS) and added to the cell cultures.

### FRET analysis

FRET based analyses were performed as previously described [40]. Twenty-four hrs after the transfection, cells were washed two times in HBSS and incubated for 30 min at 37°C. For the FRET-based time-lapse imaging, YFP emissions at 535 nm and the CFP emissions at 480 nm were recorded every 30 sec using excitation at 440 nm. Relative emission ratio was calculated using the following formula: emission ratio at each time point/emission ratio at time −1 min. The range of emission ratio was between 1.3 (maximum) and 0.6 (minimum). Emission ratio images were presented in pseudo-color in which the red range indicates high emission ratio and the blue range indicates low emission ratio. FRET efficiency was calculated using the following formula: 1 – (Fda/Fd). Fd and Fda represent donor emission intensity before and after acceptor photo-bleaching, respectively.

### Ca^2+^ imaging

Cells were loaded with Fura2-AM (1 μg, DOJINDO) at 37°C for 30 min, and then washed four times with HBSS. Fluorescence was monitored as above (FRET analysis) using specific filters (CHROMA). For time-lapse imaging, Fura2-AM emissions at 510 nm were recorded every 30 sec using alternative excitation at 340 or 380 nm, corresponding to bound and un-bound Ca^2+^ fractions, respectively. The range of 340 nm/380 nm ratio was between 0.5 (maximum) and 0.01 (minimum). 340 nm/380 nm ratio images were presented in pseudo-color in which the red range indicates high 340 nm/380 nm ratio and the blue range indicates low 340 nm/380 nm ratio.

### Western blotting

HeLa cells grown in a 35 mm dish were transiently transfected with pYFP-αCaMKII (2 μg) or pYFP-αCaMKII-CFP (2 μg). Western blotting was performed as previously described [40]. Western blot membranes were probed with anti-active αCaMKII phospho-T286 antibody (1:2000, PROMEGA), anti-αCaMKII antibody (1:2000, Santa Cruz Biotechnology) or anti-β-Actin antibody (1:2000, SIGMA) and then visualized with peroxidase-conjugated rabbit anti-mouse IgG (1:2000, Santa Cruz Biotechnology).

### Kinase assay

Kinase activity was measured as previously described [17,41]. Twenty-four hrs after the transfection, cells were homogenized in PBS on ice. Cell extracts were first pre-incubated in reaction buffer (50 mM Tris (pH 7.5), 10 mM MgCl_2_, 2 mM DTT, 0.1 mM EDTA, 100 μM ATP and 2 mM CaCl_2_) for 10 min at 37°C and then autocamtide-2, a synthetic peptide which is a specific substrate for αCaMKII, was added at final concentration of 20 μM together with 0.4 mM (200–600 cpm/pmol) of [γ-^32^P-ATP]. The reaction mixtures were incubated for 5 min at 37°C. The mixtures were characterized by spotting 15 μl of the supernatant on P-81 phosphocellulose paper (Whatman). The spotted papers were subsequently washed in 75 mM phosphoric acid. ^32^P incorporation into peptide was quantified as described using Cerenkov radiation.

### Molecular weight determinations

Gel filtration was performed as previously described [42]. COS-1 cells grown in a 100 mm dish were transiently transfected with pWT-αCaMKII (8 μg), pYFP-αCaMKII (8 μg) or pYFP-αCaMKII-CFP (8 μg). Twenty-four hrs after the transfection, whole cell extracts were fractured by lysis buffer containing 0.5% Nonidet-P40 (NP-40), 10 mM Na_2_HPO_4_ [pH 7.5], 150 mM KCl and 2 mM EDTA. Cell extracts were loaded onto a Sephacryl S-300 gel filtration column (GE Healthcare) in buffer containing 20 mM HEPES [pH 7.5], 150 mM KCl, 0.1 mM EDTA and complete EDTA-free (Roche). Thyrogloblin (669 kDa), ferritin (440 kDa), catalase (232 kDa) and aldolase (158 kDa) were used as standards for molecular weight determination. The elution positions of WT-αCaMKII or αCaMKII fusion proteins were determined from absorbance at 280 nm of the column effluent while collecting fractions. In order to confirm that the identified peaks were αCaMKII proteins, the proteins in each fraction were analyzed by Western blotting.

## Competing interests

The authors declare that they have no competing interests.

## Authors’ contributions

SK is responsible for the hypothesis development and overall design of the research and experiment, and supervised the experimental analyses. SK and KK co-wrote the manuscript. KK performed all experiments. TI participated in the cloning of the expression plasmids and design. All authors read and approved the final manuscript.

## Supplementary Material

Additional file 1 **Figure S1. (A)** Long-term imaging in HeLa cells expressing αCaMKII fusion proteins at 30 sec intervals for 25 min. Upper graph shows the interaction of YFP-αCaMKII with CFP-CaM. Lower graph shows conformational change of YFP-αCaMKII-CFP. **(B)** Short-term imaging in HeLa cells expressing αCaMKII fusion proteins at 1 sec intervals for 1.5 min. Upper graph shows the interaction of YFP-αCaMKII with CFP-CaM. Lower graph shows the conformational change of YFP-αCaMKII-CFP. Black bars shows the period of ionomycin application (A and B). 5–10 cells were analyzed in each group. **(C)** Imaging of changes in Ca^2+^ concentration using Fura2-AM when HBSS perfusion was performed at 1 min following ionomycin application. 10–20 cells were analyzed in each group. Black bar and white bar show the period of ionomycin application. 10–15 cells were analyzed in each group. Lower panels show auto-phosphorylation at T286 of YFP-αCaMKII-CFP following ionomycin application at each time point (0, 1, 5 and 10 min) with or without of removal of ionomycin. Black bar and white bar show the period of ionomycin application. Click here for file

Additional file 2 **Figure S2.** Expression levels of αCaMKII fusion proteins in HeLa cells. **(A)** Western blot analysis of the expression level of αCaMKII fusion proteins. Left panel shows expression level of YFP-αCaMKIIs and CFP-CaM (black arrow head, YFP-αCaMKIIs; white arrow head, CFP-CaM). Right panel shows expression level of YFP-αCaMKII-CFPs (black arrow head, YFP-αCaMKII-CFPs). **(B)** Effects of ionomycin application upon the expression levels of αCaMKII fusion proteins. Western blot analyses analyzing the expression level of αCaMKII fusion proteins were performed using GFP specific antibody (black arrow head, YFP-αCaMKIIs or YFP-αCaMKII-CFPs; white arrow head, CFP-CaM). β-Actin specific antibody was used as a loading control. Click here for file
