# Supplementary figures and images for: Interactions between αCaMKII and calmodulin in living cells: conformational changes arising from CaM -dependent and -independent relationships
Source: Mol Brain. 2013 Aug 19;6:37. doi: 10.1186/1756-6606-6-37 (PMC3765210; doi:10.1186/1756-6606-6-37)

**A**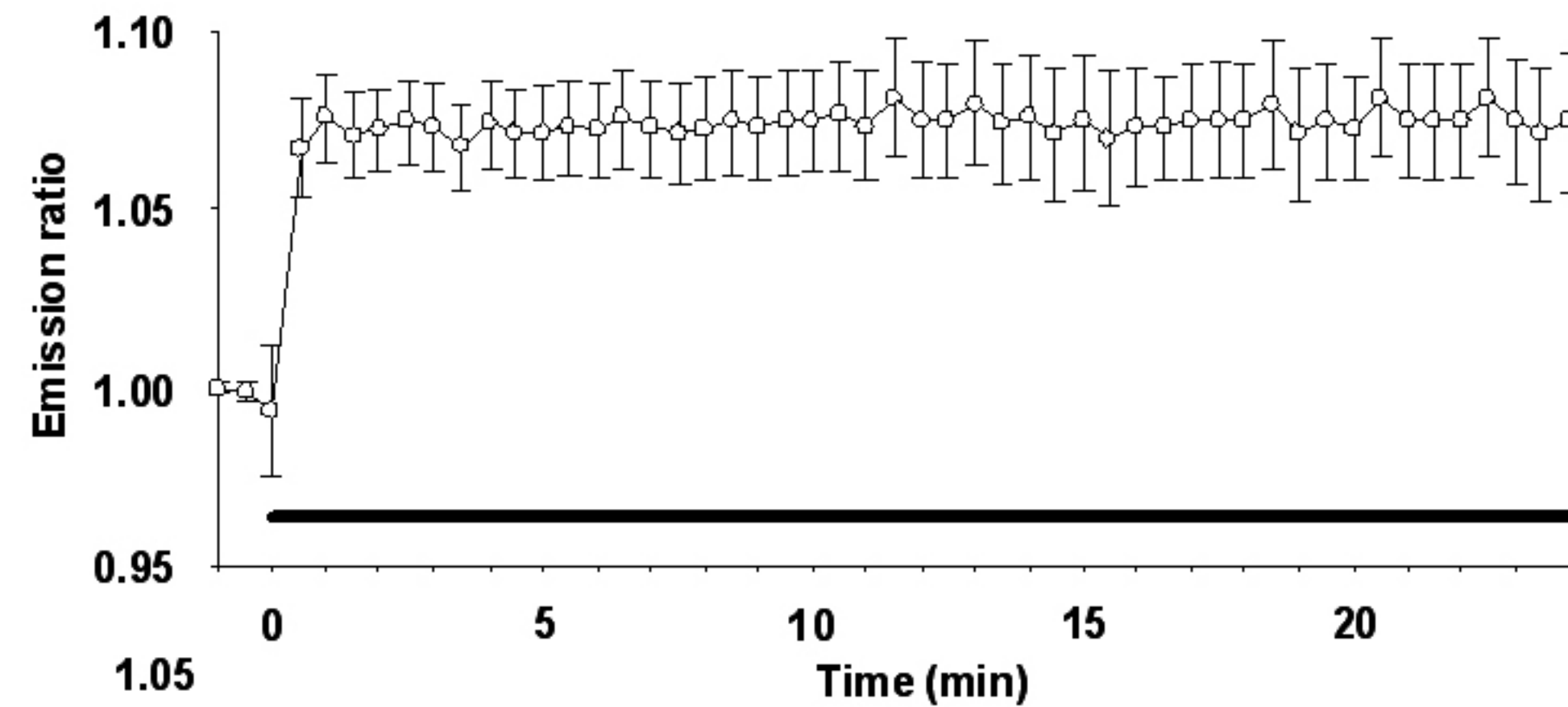**B**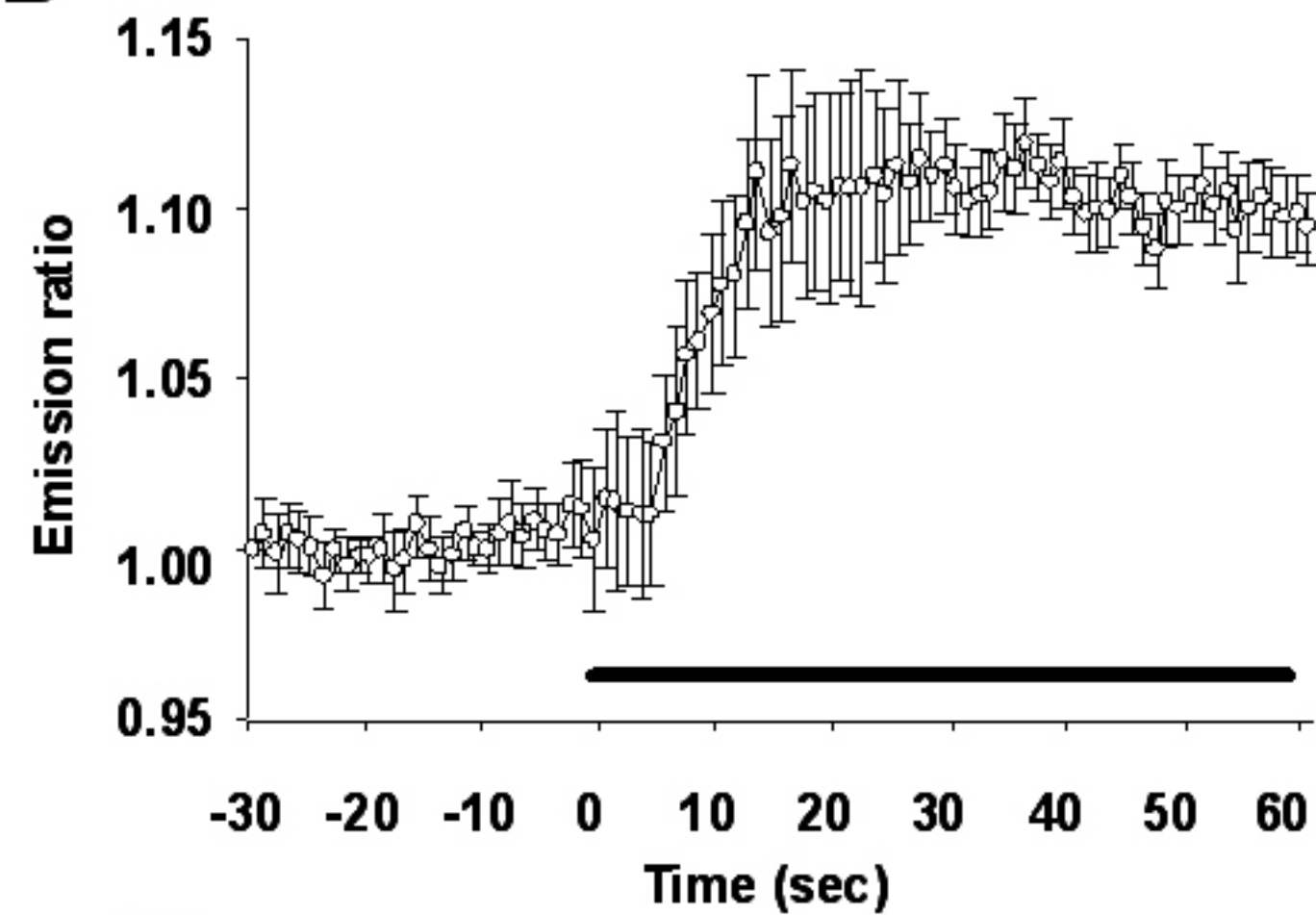**C**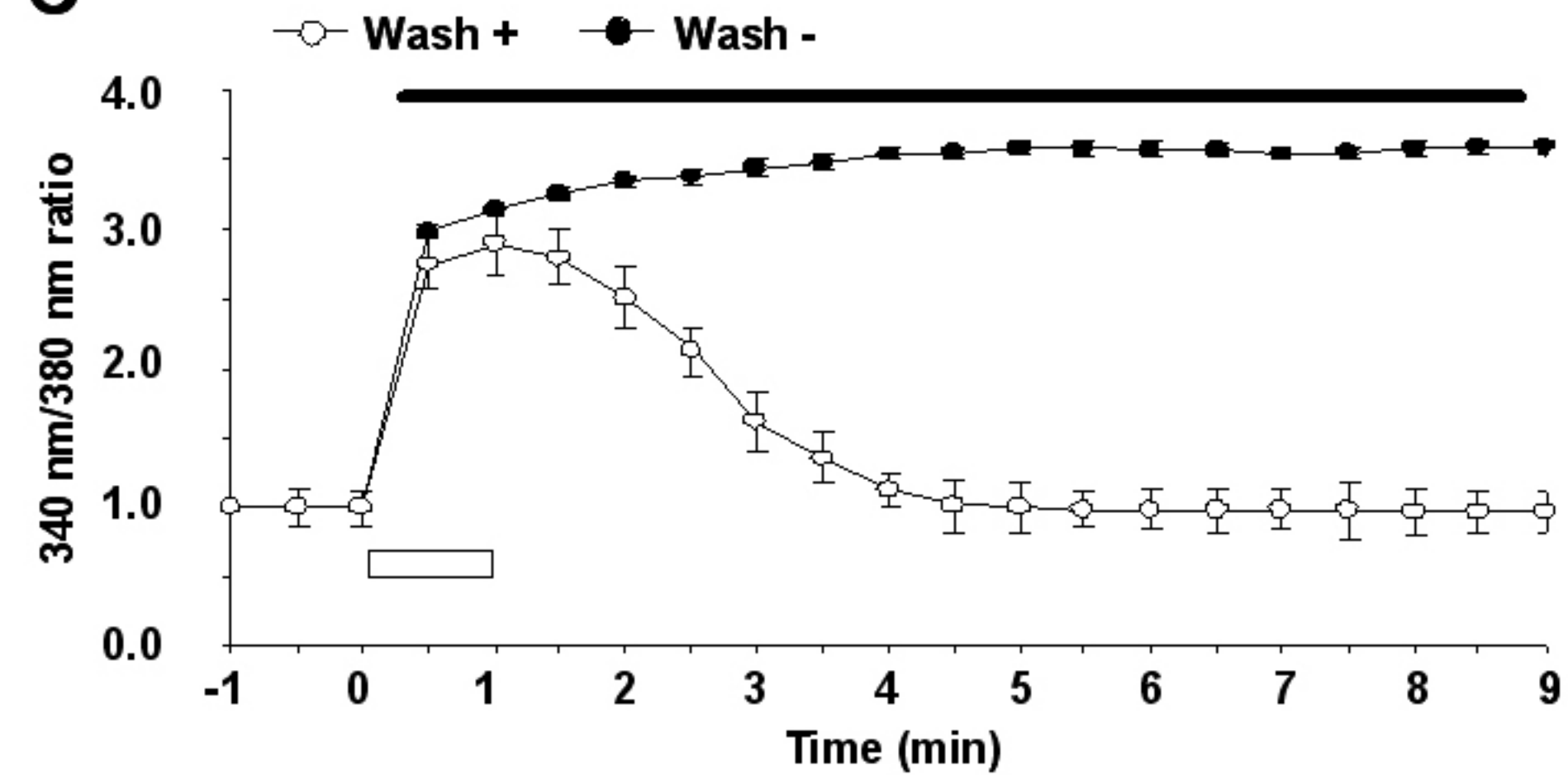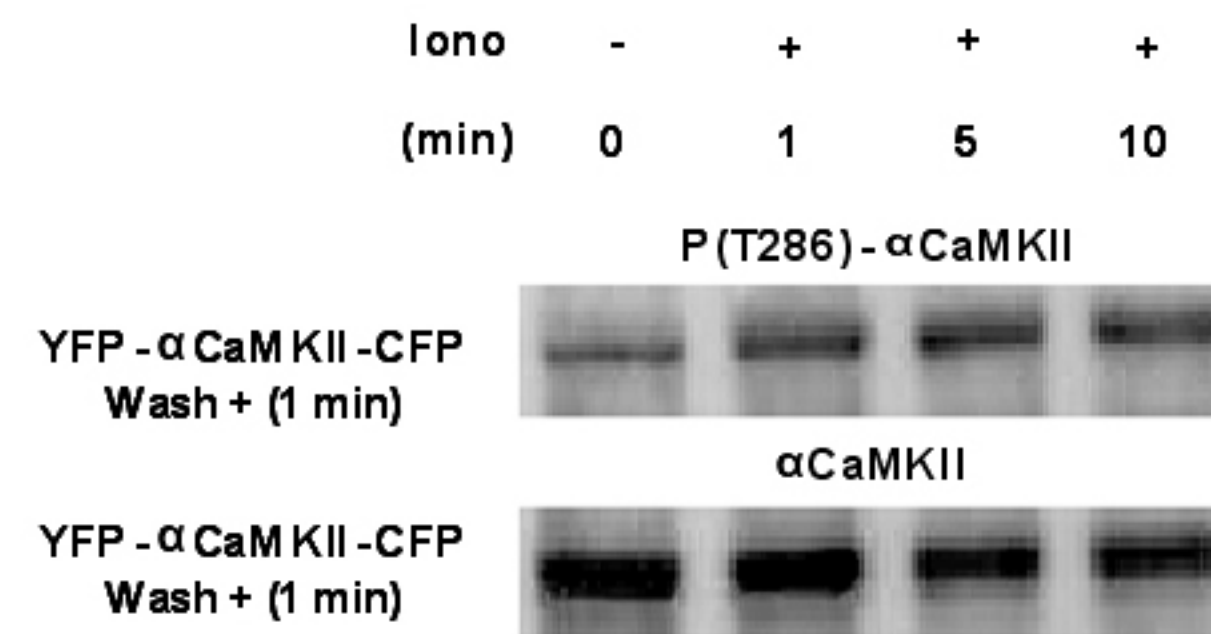

Supplement: Additional file 1 — Figure S1. (A) Long-term imaging in HeLa cells expressing αCaMKII fusion proteins at 30 sec intervals for 25 min. Upper graph shows the interaction of YFP-αCaMKII with CFP-CaM. Lower graph shows conformational change of YFP-αCaMKII-CFP. (B) Short-term imaging in HeLa cells expressing αCaMKII fusion proteins at 1 sec intervals for 1.5 min. Upper graph shows the interaction of YFP-αCaMKII with CFP-CaM. Lower graph shows the conformational change of YFP-αCaMKII-CFP. Black bars shows the period of ionomycin application (A and B). 5–10 cells were analyzed in each group. (C) Imaging of changes in Ca2+ concentration using Fura2-AM when HBSS perfusion was performed at 1 min following ionomycin application. 10–20 cells were analyzed in each group. Black bar and white bar show the period of ionomycin application. 10–15 cells were analyzed in each group. Lower panels show auto-phosphorylation at T286 of YFP-αCaMKII-CFP following ionomycin application at each time point (0, 1, 5 and 10 min) with or without of removal of ionomycin. Black bar and white bar show the period of ionomycin application. [file 1756-6606-6-37-S1.pdf]
