# Supplementary material for: Interactions between αCaMKII and calmodulin in living cells: conformational changes arising from CaM -dependent and -independent relationships
Source: Mol Brain. 2013 Aug 19;6:37. doi: 10.1186/1756-6606-6-37 (PMC3765210; doi:10.1186/1756-6606-6-37)
Supplement: Additional file 2 — Figure S2. Expression levels of αCaMKII fusion proteins in HeLa cells. (A) Western blot analysis of the expression level of αCaMKII fusion proteins. Left panel shows expression level of YFP-αCaMKIIs and CFP-CaM (black arrow head, YFP-αCaMKIIs; white arrow head, CFP-CaM). Right panel shows expression level of YFP-αCaMKII-CFPs (black arrow head, YFP-αCaMKII-CFPs). (B) Effects of ionomycin application upon the expression levels of αCaMKII fusion proteins. Western blot analyses analyzing the expression level of αCaMKII fusion proteins were performed using GFP specific antibody (black arrow head, YFP-αCaMKIIs or YFP-αCaMKII-CFPs; white arrow head, CFP-CaM). β-Actin specific antibody was used as a loading control. [file 1756-6606-6-37-S2.pdf]

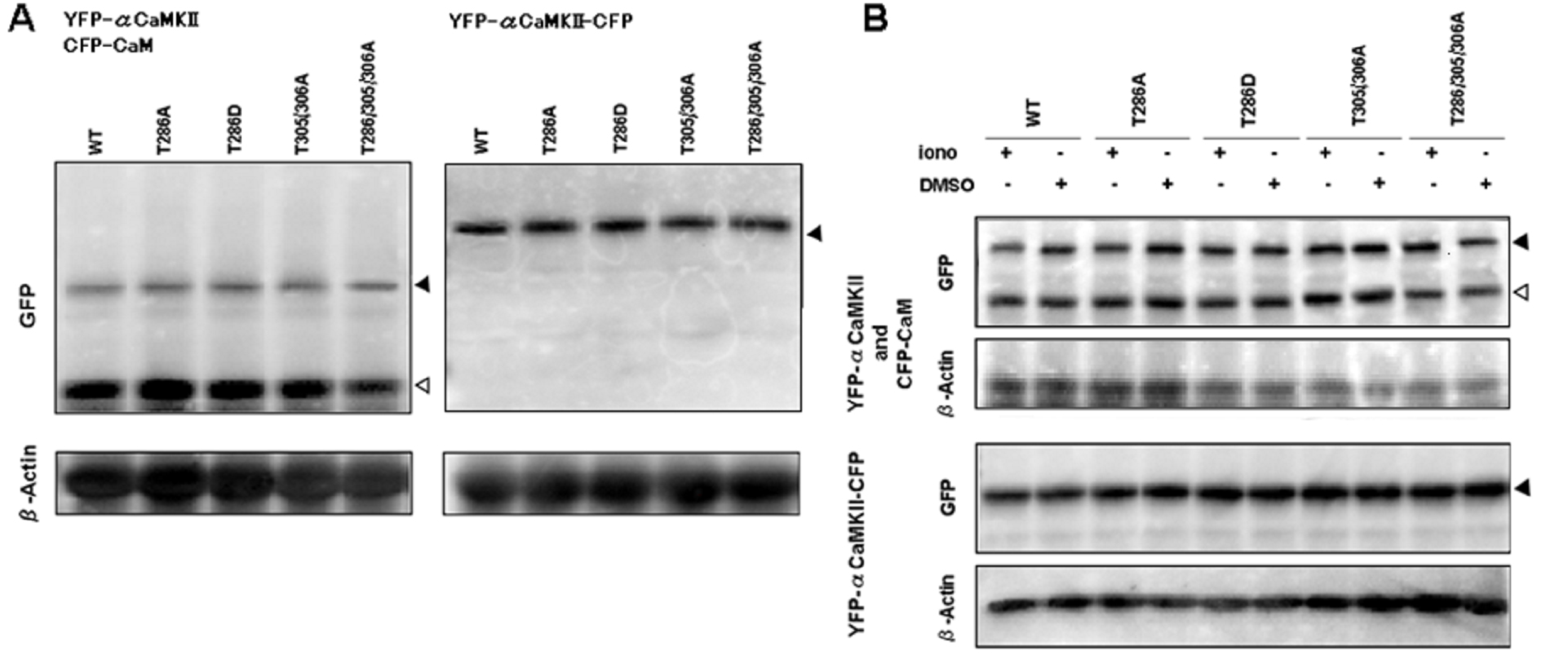
A
YFP-αCaMKII
CFP-CaM
YFP-αCaMKII-CFP
WT
T286A
T286D
T305/306A
T286/305/306A
GFP
β-Actin
B
iono
DMSO
YFP-αCaMKII and CFP-CaM
YFP-αCaMKII-CFP
